# Human Hepatitis B Virus Production in Avian Cells Is Characterized by Enhanced RNA Splicing and the Presence of Capsids Containing Shortened Genomes

**DOI:** 10.1371/journal.pone.0037248

**Published:** 2012-05-18

**Authors:** Josef Köck, Christine Rösler, Jingjing Zhang, Hubert E. Blum, Michael Nassal, Christian Thoma

**Affiliations:** Department of Medicine II, University Hospital of Freiburg, Freiburg, Germany; Yonsei University, Korea

## Abstract

Experimental studies on hepatitis B virus (HBV) replication are commonly done with human hepatoma cells to reflect the natural species and tissue tropism of the virus. However, HBV can also replicate, upon transfection of virus coding plasmids, in cells of other species. In such cross-species transfection experiments with chicken LMH hepatoma cells, we previously observed the formation of HBV genomes with aberrant electrophoretic mobility, in addition to the those DNA species commonly seen in human HepG2 hepatoma cells. Here, we report that these aberrant DNA forms are mainly due to excessive splicing of HBV pregenomic RNA and the abundant synthesis of spliced DNA products, equivalent to those also made in human cells, yet at much lower level. Mutation of the common splice acceptor site abolished splicing and in turn enhanced production of DNA from full-length pgRNA in transfected LMH cells. The absence of splicing made other DNA molecules visible, that were shortened due to the lack of sequences in the core protein coding region. Furthermore, there was nearly full-length DNA in the cytoplasm of LMH cells that was not protected in viral capsids. Remarkably, we have previously observed similar shortened genomes and non-protected viral DNA in human HepG2 cells, yet exclusively in the nucleus where uncoating and final release of viral genomes occurs. Hence, two effects reflecting capsid disassembly in the nucleus in human HepG2 cells are seen in the cytoplasm of chicken LMH cells.

## Introduction

Hepatitis B virus (HBV) primarily infects humans and virus amplification takes place exclusively in the liver. To reflect this tight species and tissue tropism, studies of HBV replication are commonly done with human HepG2 or HuH-7 hepatoma cells [1], [2]. Both cell lines are not susceptible to HBV infection but they support the synthesis of virus particles upon transfection of viral genome containing plasmids. Assembly of HBV capsids occurs in the cytoplasm and starts with packaging of viral polymerase and pregenomic RNA (pgRNA) by newly made core proteins. Inside the capsid, the viral polymerase converts pgRNA into single stranded DNA. This reverse transcription initiates from a specific tyrosine residue in the polymerase protein, which thereby becomes covalently linked to the 5′-end of nascent minus-strand DNA [3], [4], [5]. Subsequent synthesis of the complementary second strand results in the formation of relaxed circular DNA (rcDNA) in which both strands partially overlap, and a fraction of double strand linear DNA which arises if circle formation does not properly proceed [6], [7]. Furthermore, some capsids contain smaller genomes that originate from reverse transcription of various spliced forms of pgRNA [8], [9]. Such capsids containing spliced genomes are usually present at low frequency compared to those with full-length DNA.

Much of our knowledge of the HBV life cycle has been obtained from comparative studies with the distantly related duck hepatitis B virus (DHBV) which is endemic in ducks [10], [11]. DHBV replication is commonly studied in the chicken liver tumour derived LMH cell line [12], [13]. DHBV capsids made in the cytoplasm of transfected LMH cells are either secreted into culture supernatant or transported to the cell nucleus where the viral genome is released and converted into covalently closed circular DNA (cccDNA) [14]. Such intracellular recycling of viral genomes also occurs in HepG2 cells that are transfected with HBV coding plasmid. The efficiency of cccDNA formation in HBV producing HepG2 cells, however, is much lower than in DHBV synthesizing LMH cells. Human cells instead accumulate rcDNA in the nucleus that is released from the capsid and detached from the polymerase protein [15], [16], [17]. In addition, there are HBV capsids in the nucleus of HepG2 cells that contain genomes, which are shortened and lack sequences in the core protein coding region. Furthermore, HepG2 cell nuclei harbour almost full-length DNA that is not protected in viral capsids but is nevertheless connected with polymerase protein.

To define the contribution of virus versus host cell in the genome recycling pathway, we have previously performed cross-species transfection experiments in which DHBV was expressed in human HepG2 and HBV in chicken LMH cells. We found that DHBV produces high amounts of cccDNA not only in LMH cells but also in HepG2 cells. On the other hand, the cccDNA levels of HBV were low, both in HepG2 and in LMH cells [17]. Thus, low-level genome recycling is an intrinsic feature of HBV, rather than a property of the cell line in which the virus replicates. During our previous investigation, we further noticed that HBV capsids from cytoplasm of LMH cells contain genomes with cccDNA-like electrophoretic mobility, in addition to those DNA forms that are commonly seen in HepG2 cells. As cccDNA is not expected to be present in viral capsids, we sought to characterize this particular DNA in more detail. Here, we report that the respective genomes are not cccDNA, but instead 2.0 kb double strand linear molecules derived from spliced transcripts. We show that LMH cells produce exceptionally high amounts of spliced RNA, explaining the high abundance of splicing-derived DNA in HBV capsids from the avian cell line. Sequencing of two additional, less prominent splicing products revealed them to be identical with those previously found in human cells, and mutation of the common splice acceptor site abolished splicing in HepG2 and LMH cells. The pattern of viral DNA obtained in the avian cell line, was nevertheless different, and many of the HBV particles from cytoplasm of LMH cells contained shortened genomes that are not derived from spliced RNA and lack sequences in the core protein coding region. In this respect, they are similar to those genomes previously discovered in the nucleus of HepG2 cells. In addition, there were almost full-length genomes in cytoplasm of LMH cells that are connected with polymerase protein but not protected in viral capsids. Thus, two features of HBV replication that appear to be confined to the nucleus of HepG2 cells are found in the cytoplasm of LMH cells.

## Materials and Methods

### Cell culture and transfection

Chicken LMH cells and human HepG2 cells (both obtained from American Type Culture Collection) were cultured in Iscove's Modified Dulbecco's Medium (Gibco) supplemented with 10% foetal calf serum, 100 U/ml of streptomycin and 100 µg/ml penicillin. Culture dishes were coated with rat-tail collagen (BD Biosciences) which facilitates more uniform growth and distribution of HepG2 cells. To assure identity of the cell lines, we confirmed their species origin by amplification and partial sequencing of the mitochondrial 16S rRNA genes. Furthermore, short tandem repeat authentication of HepG2 chromosomal DNA was done, yielding a profile identical to the one listed by the American Type Culture Collection. Transfection was done with 6 µl of TransIT-LT1 liposomal reagent (Mirus Bio) and 2 µg of plasmid DNA per well of a 6 well culture dish.

The plasmids, applied in this study, are derived from HBV subtype *ayw* and harbour an over-length genome, which enables transcription of authentic pgRNA either from the endogenous core promoter or from the CMV promoter [18], [19], [20]. To abrogate splicing of HBV transcripts, the adenine residue at position 1769 relative to the core start codon was replaced by cytosine. This A1769C mutation does not change the amino acid sequence of the viral polymerase, and has no effect on hepatitis B surface protein, which is encoded on a second, overlapping reading frame. Chicken LMH cells do not synthesize detectable levels of surface protein coding RNA. Thus, transfected LMH cells do not secrete enveloped HBV particles. To allow for comparison with HepG2 cells, which otherwise secrete rcDNA containing capsids into culture supernatant, we introduced two additional nucleotide substitutions (C1399A and T1438C) which prevent the synthesis of surface proteins. These two mutations do not alter the polymerase protein and have no effect on splicing of pgRNA [21]. The plasmids were grown in *E.coli* strain, positive for Dam-methylation to enable digestion of plasmid DNA with restriction enzyme *Dpn*I.

### Preparation of viral DNA and RNA

Three days after transfection, the cells were detached by trypsin treatment, resuspended in culture medium and collected by centrifugation. The cell pellets were resuspended in 0.5 ml of lysis buffer (140 mM NaCl, 1.5 mM MgCl_2_, 50 mM Tris-HCl [pH 8.0] and 0.5% Nonidet P-40). Cell nuclei were subsequently removed by centrifugation and the cytoplasm containing supernatant was treated with 30 units of micrococcal nuclease (New England BioLabs) for 2 hours at 37°C in the presence of 2 mM CaCl_2_. Then, micrococcal nuclease was inactivated by adding EDTA to a final concentration of 5 mM and the sample was digested with 0.5 mg/ml Proteinase K in the presence of 0.5% SDS for 1 hour at 56°C. Afterwards, RNase A (10 µg/ml) was added and viral DNA was extracted twice with Tris-buffered phenol/chloroform/isoamyl alcohol (25/24/1) and once with chloroform. Precipitation was done with ethanol and the DNA was finally dissolved in 50 µl of 10 mM Tris-HCl [pH 8.0]. The DNA samples were treated with Plasmid safe DNase (Epicentre Biotechnologies), or digested with restriction enzyme *Dpn*I (New England Biolabs) or denatured by heating at 95°C for 5 minutes, as indicated in the respective experiments.

For complete removal of cytoplasmic capsids, HepG2 cell nuclei were purified by sucrose gradient centrifugation. In brief, the cells were harvested as described above and dissolved in 4.0 ml of low sucrose buffer (0.32 M sucrose, 3 mM CaCl_2_, 2 mM magnesium acetate, 1 mM dithiothreitol, 10 mM Tris-HCl [pH 8.0], 0.5% Nonidet P-40). Subsequently, the cell lysate was gently mixed with 4.0 ml of high sucrose buffer (2.0 M sucrose, 5 mM magnesium acetate, 1 mM dithiothreitol, 10 mM Tris-HCl [pH 8.0]), and loaded on top of a cushion made of 4.4 ml high sucrose buffer. All these preparation steps were done at 4°C. Centrifugation was performed for 45 minutes with 15,500 rpm (44,000×g) at 4°C in a Kontron swing-out TST41 rotor. The supernatant was subsequently removed by aspiration and cell nuclei at the bottom of the tube were resuspended in 0.4 ml lysis buffer (140 mM NaCl, 1.5 mM MgCl_2_, 50 mM Tris-HCl [pH 8.0], 0.5% Nonidet P-40). This suspension was mixed with 30 units of micrococcal nuclease and incubated for 6 hours at 37°C in the presence of 2 mM CaCl_2_ before DNA extraction was done, as described above.

RNA was prepared from HepG2 and LMH cells with the RNeasy Mini columns from Qiagen. Treatment with RNase free DNase was included during RNA preparation to remove viral DNA and transfected plasmid. The RNA samples were separated on 1% agarose gels containing 2% formaldehyde. The gel was subsequently soaked in 50 mM NaOH for 20 minutes to improve transfer of large transcripts. Northern blot hybridisation was done with a ^32^P-labeled fragment obtained by digestion of HBV coding plasmid with restriction enzyme *Bgl*II, which cuts at position 84 and 523 relative to start codon the core gene.

### Southern blot detection and PCR analysis

DNA samples were loaded onto TAE-buffered 1.4% low EEO agarose gels (BioRad). A mixture of the 3.2 kb *Eco*RI fragment with the 2.0 kb and 1.2 kb *Eco*RI/*Sph*I fragments of the HBV genome served as size markers. Alternatively, the 2.7 kb pUC19 vector plasmid containing a 0.5 kb *Eco*RI/*Bam*HI fragment of the HBV genome was applied as marker for 3.2 kb circular genomes. Southern blots were probed with a mixture of the 1.4 kb *Eco*RI/*Nco*I and the 1.8 kb *Nco*I/*Eco*RI fragment obtained from an HBV coding plasmid. These two fragments were separated from vector sequences by agarose gel electrophoresis and isolated from gel slices. The corresponding probe detects both strands of the whole HBV genome and likewise visualizes viral DNA derived from spliced and full-length pgRNA. This probe visualizes the double-stranded forms of the HBV genome as well as single stranded precursor molecules.

Alternatively, hybridisation was done with ^32^P-labeled oligonucleotides 17 reverse (5′-TAAGGGTCGATGTCCATGCCC-3′), 18 forward (5′-AAAGAATTTGGAGCTACTGTGGAG-3′), 172 forward (5′-GCAATTCTTTGCTGGGG-3′), 355 forward (5′-TTGGTGTCTTTCGGAGTGTG-3′), 399 forward (5′-ACCACCAAATGCCCCTATCC-3′), 470 forward (5′-GAAGAAGAACTCCCGCGCCTCGCAGACGAAG-3′), 520 forward (5′-AGAAGATCTCAATCTCCGGAATCTC-3′), 2307 reverse (5′-GCAGCAAAACCCAAAAGACCC-3′), 2340 reverse (5′-GGCATCAACGCAGGATAACCA-3′), 2385 reverse (5′-GGCGAGAAAGTGAAAGCCTGC-3′), 2439 reverse (5′-CGGGCAACGGGTAAAGGTTCAGG-3′), 2682 reverse (5′-CAGTTGGCAGCACAGCCTAGCAGC-3′).

PCR amplification of viral DNA was done with primers 18 forward (5′-AAAGAATTTGGAGCTACTGTGGAG-3′) and 2855 reverse (5′-CCGGCAGATGAGAAGGCACAGACCCC-3′). Twenty cycles were performed with elongation at 72°C lasting for 3 minutes to favour the synthesis of large fragments. The PCR fragment, arising form the predominant spliced HBV genome species, commonly termed Sp1, was cut out from the agarose gel and subjected to direct sequencing. Another aliquot of the PCR products, obtained with primers 18 forward and 2855 reverse, was digested with restriction enzyme *Bsp*EI, which cuts at genome position 429. Fragments derived from Sp3 spliced species lack the *Bsp*EI restriction site and therefore remain complete after *Bsp*EI digestion. These non-digested fragments were further amplified by semi-nested PCR with primers 18 forward and 2439 reverse (5′-CGGGCAACGGGGTAAAGGTTCAGG-3′) before direct sequence analysis.

Alternatively, viral DNA from transfected LMH cells was digested with restriction enzyme *Eco*RI, which cuts at genome position 1280, and subsequently treated with Plasmid safe DNase. Thus, Plasmid safe DNase removes single stranded precursor genomes, *Eco*RI digested full-length rcDNA, full-length linear and spliced linear genomes, but not the Sp14 spliced species, which lacks the *Eco*RI site and forms circular molecules [22]. Amplification was done with primer 18 forward and primer 2855 reverse, followed by nested PCR with primer 556 forward (5′-TCCTTGGACTCATAAGGTGGG-3′) and primer 2439 reverse. The resultant PCR fragment was subjected to direct sequence analysis.

## Results

### HBV particles from transfected LMH cells contain high amounts of spliced genomes

**Figure 1:**
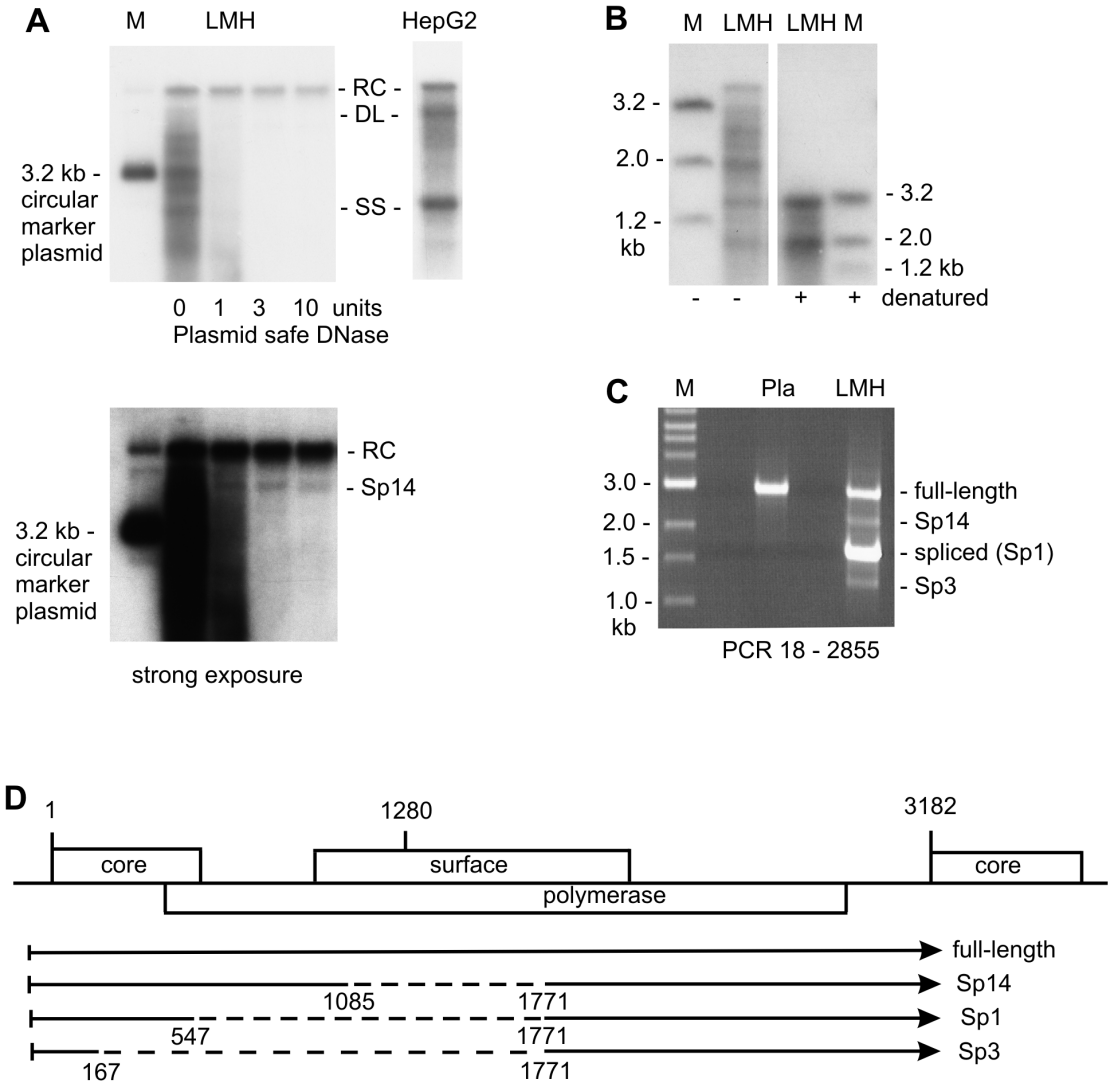
Identification of spliced HBV genomes in transfected LMH cells. The plasmid transfected is coding for wild type HBV with the natural core promoter driving transcription of pgRNA. Viral DNA was prepared from cell cytoplasm that was treated with micrococcal nuclease to remove the transfected plasmid. (**A**) Viral DNA from transfected LMH cells was incubated with increasing amounts of Plasmid safe DNase and visualized by Southern blot analysis using a ^32^P-labelled probe covering both strands of the whole HBV genome. A circular 3.2 kb plasmid, which migrates at the position of cccDNA, served as marker (M). Positions of relaxed circular (RC), double strand linear (DL) and single stranded (SS) viral DNA are indicated. Viral DNA from cytoplasm of transfected HepG2 cells is shown as reference. The lower panel is a strong exposure of the blot to visualize the Sp14 spliced DNA which is resistant to Plasmid safe DNase digestion. (**B**) Viral DNA from transfected LMH cells and linear marker fragments were denatured by heating before gel electrophoresis. The left panel shows non-denatured DNA for comparison. The positions of linear size marker (M) fragments are indicated. (**C**) Viral DNA was amplified with primers binding at genome position 18 and 2855 relative to the core start codon. Plasmid (Pla) containing the full-length HBV genome served as reference template. Amplification products derived from full-length HBV genome and the predominant spliced species (Sp1) are indicated. Less prominent amplification products, representing Sp3 and Sp14 spliced species, are also indicated. (**D**) The diagram shows a map of the HBV genome with the core, polymerase and surface coding sequences drawn as rectangles. The core gene is presented twice to indicate that the viral genome has a circular structure. The start codon of the core protein coding gene is position 1 in the here applied nomenclature with the whole HBV genome consisting of 3182 nucleotides. At genome position 1280, there is an *Eco*RI restriction site, which serves as point of reference in other commonly applied nomenclature. Arrows below symbolize spliced and full-length pgRNA. The dashed lines illustrate the intron sequences that are removed in Sp14, Sp1 and Sp3 spliced species. The corresponding splice donor sites are at position 167, 547 and 1085 while the common splice acceptor site is at position 1771. If numbering starts with the *Eco*RI restriction site, the common splice acceptor site will be position 487 and the splice donor sites will be position 2065, 2445 and 2983, according to Abraham *et al.*, 2008.

Viral DNA made in HBV capsids upon transfection of HepG2 cells with virus coding plasmid mainly consists of single stranded, double strand linear and relaxed circular molecules. These DNA forms are also found in HBV capsids from cytoplasm of transfected LMH cells, however together with various additional DNA species (Figure 1A). Among those, the most prevalent co-migrates with a 3.2 kb super helical plasmid, which served as a marker for cccDNA in our previous investigation. To test whether this particular DNA has a circular structure, we treated viral DNA from cytoplasm of LMH cells with Plasmid safe DNase, an enzyme that selectively digests single strand and double strand linear DNA but not double strand circular molecules. As expected, the rcDNA was completely resistant to Plasmid safe DNase digestion. Other nucleic acids, including those molecules co-migrating with the 3.2 kb marker plasmid, disappeared when incubated with increasing amounts of the enzyme (Figure 1A). Thus, we conclude that the novel DNA species, abundantly present in HBV capsids from transfected LMH cells, is not of circular structure.

Next, we compared the electrophoretic mobility of viral DNA from LMH cells with that of linear marker fragments. This analysis revealed that the predominant DNA species co-migrates with a 2.0 kb restriction fragment (Figure 1B, left panel). Denaturation of the sample before gel loading disclosed two major viral DNA forms: the first co-migrated with the denatured 3.2 kb marker fragment, the second with the denatured 2.0 kb marker fragment (Figure 1B, right panel). This result is consistent with HBV capsids from LMH cells containing primarily 3.2 kb and 2.0 kb viral genomes. The more complex pattern seen in the non-denatured sample is due to the presence of single stranded precursor forms of the 3.2 kb and 2.0 kb genomes. In addition, there is a signal migrating between the 3.2 kb and 2.0 kb marker fragments. This particular DNA species will be discussed in more detail, later.

We reasoned that the 2.0 kb species might be a spliced variant of the HBV genome. Therefore, we performed PCR analysis with primers spanning almost the entire HBV genome. The main amplification product obtained with viral DNA from LMH cells was significantly smaller than the reference fragment amplified from cloned DNA (Figure 1C). Sequence analysis revealed that the PCR product was derived from a viral genome in which a 1.2 kb fragment, ranging from position 547 to position 1771 relative to the core start codon, was deleted (see map of the HBV genome in Figure 1D). Such 2.0 kb genomes are known to be generated also in human cells if a spliced variant of pgRNA, commonly termed Sp1, is encapsidated and converted into DNA [22], [23].

Selective amplification and sequence analysis of two additional faint PCR signals, visible in Figure 1C, revealed that these products are derived from spliced genomes in which the donor sites localize to position 167 and 1085, respectively, with the common acceptor site being at position 1771 (see map of the HBV genome in Figure 1D). The same spliced genomes, termed Sp3 and Sp14, also exist in human cells [22], [23]. The Sp14 species has been reported to generate a circular molecule, which is expected to resist Plasmid safe DNase digestion. A faint signal, most likely representing the Sp14 species, was indeed visible upon strong exposure of the Southern blot of Figure 1A, see lower panel. These data indicate that pgRNA splicing, as such, is similar in human and avian cells, but in LMH cells, there is either preferential packaging of spliced transcripts or the extent of RNA splicing itself is higher than in human cells, especially for the Sp1 species.

### Enhanced splicing of pgRNA in avian cells gives rise to abundant spliced viral DNA

The CAGG consensus sequence at position 1768 to 1771 in the HBV genome represents a common splice acceptor site, shared by various splicing products. We reasoned that mutating this site should greatly reduce splicing and in turn promote the formation of full-length genomes in LMH cells. To test the assumption, we replaced the adenine residue at position 1769 in the HBV genome for a cytosine residue (A1769C). The acceptor site in the HBV genome localizes to the middle of the polymerase-coding gene. Importantly, the A1769C mutation does not change the amino acid sequence and function of polymerase. According to a previous report, splicing levels in human hepatoma cells strongly depend on the promoter driving transcription of pgRNA [22]. To assess for similar promoter effects in avian cells, we additionally tested a splicing-competent HBV plasmid in which the endogenous core promoter was substituted by the immediate early promoter of cytomegalovirus (CMV).

**Figure 2:**
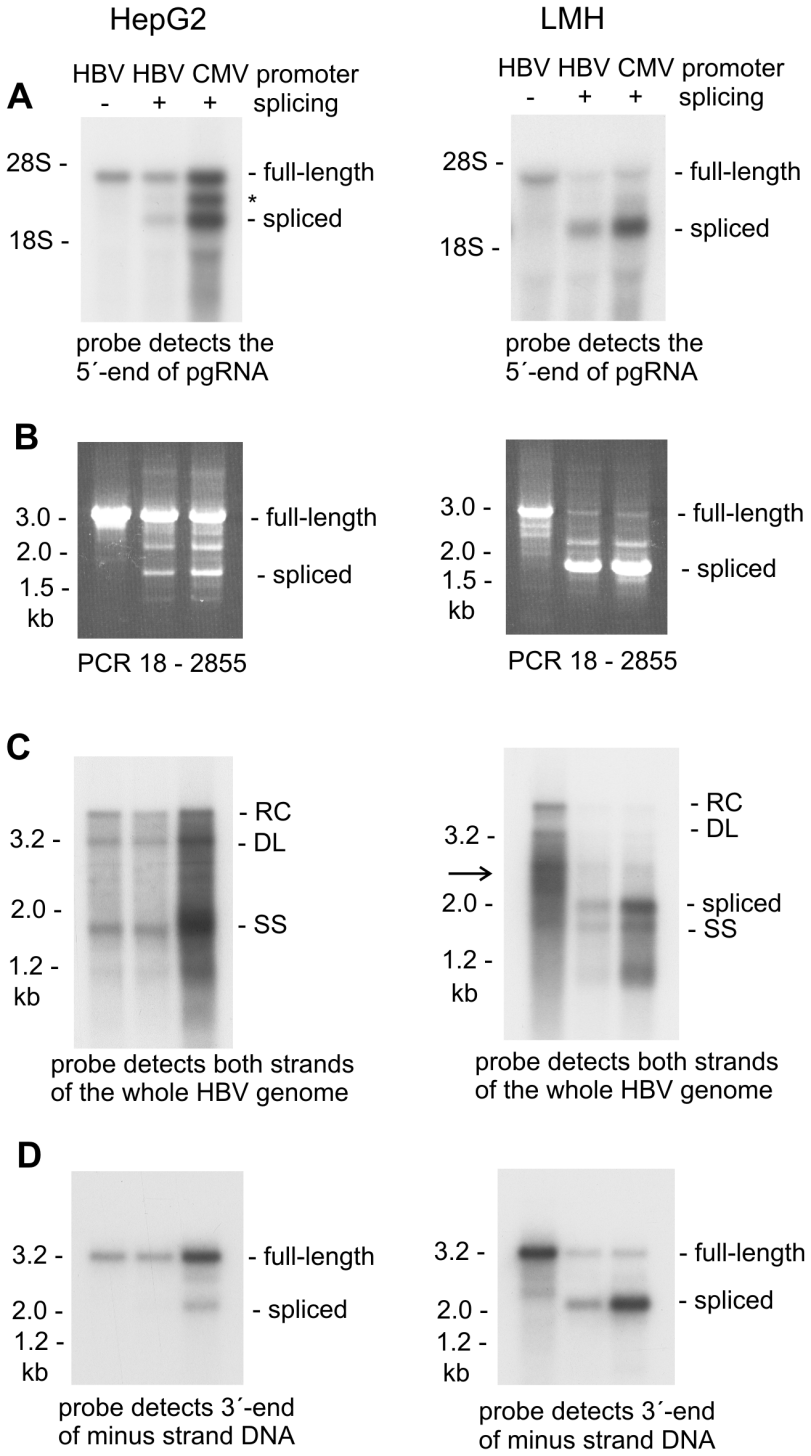
Comparison of HBV replication in human HepG2 and chicken LMH cells. The transfected plasmids are coding for splice-deficient and splicing-competent virus (+/− splicing) with either the natural HBV core promoter or the CMV promoter driving transcription of pgRNA. The here applied plasmids additionally have the surface protein coding sequence mutated to abrogate secretion of rcDNA containing particles in human HepG2 cells. (**A**) Viral RNA from cytoplasm of HepG2 and LMH cells was visualized by Northern blot analysis. Hybridization was done with a probe specific to the 5′-end of spliced and full-length pgRNA (genome position 84 to 523). This probe corresponds to the core protein coding sequence (see map of Figure 1D) and does not detect surface protein coding transcripts. The 28S and 18S ribosomal RNA served as size marker. Positions of full-length pgRNA and predominant Sp1 spliced species are indicated. The asterisk denotes another transcript probably representing Sp14 spliced RNA abundantly made in HepG2 cells. (**B**) Viral DNA from transfected HepG2 and LMH cells was amplified with primers spanning the intron sequence in the HBV genome. PCR products derived from full-length genomes and the predominant Sp1 spliced species are indicated. (**C**) Viral DNA was visualized by Southern blot hybridisation with a probe that covers both strands of the whole HBV genome, and likewise detects viral DNA derived from spliced and full-length pgRNA. Positions of relaxed circular (RC), double strand linear (DL), single stranded (SS) and spliced viral DNA are indicated. The arrow points to viral DNA migrating between the 3.2 kb and 2.0 kb marker fragments. Note that such DNA is also present in splicing-competent HBV and CMV promoter plasmid transfected LMH cells, albeit scarcely visible in the here presented exposure. The corresponding signal, however, is more clearly seen in previous Figure 1B. (**D**) Viral DNA was denatured by heating before electrophoresis and visualized by hybridisation with a mixture of ^32^P-labelled forward oligonucleotides that are complementary to the 3′-end region of minus-strand DNA (genome position 18, 172, 355, 399, 470 and 520 in the core protein coding sequence; see map of Figure 1D). Positions of full-length and Sp1 spliced genomes are indicated.

First, we compared the relative amounts of spliced versus full-length pgRNA in HepG2 and LMH cells. To this end, we performed Northern blot analysis, using a probe specific to the 5′-end of pgRNA. According to scanning of the Northern blot signals by phosphor imager, the amount of full-length pgRNA is about three times higher in CMV compared to HBV promoter plasmid transfected HepG2 cells (Figure 2A, left panel). Spliced transcripts were detectable in HepG2 cells that were transfected with splicing-competent HBV promoter plasmid, but the corresponding signal was at least two times weaker than the signal of full-length pgRNA. Transfection of the CMV promoter plasmid, however, strongly increased splicing levels in the human cell line and made the spliced transcripts slightly more abundant than full-length pgRNA. This result is accordance to a report recently published by Abraham *et al.*, 2008.

LMH cells, by comparison, produced low amounts of full-length pgRNA, if transfected with splicing-competent HBV and CMV promoter plasmid, but the amount of full-length pgRNA increased by a factor of two upon transfection of splicing-deficient HBV promoter plasmid (Figure 2A, right panel). Spliced RNA was strongly present in splicing-competent HBV promoter plasmid transfected LMH cells (more than twice as much as full-length pgRNA), and was about five times more abundant than full-length pgRNA in CMV promoter plasmid transfected cells. Thus, chicken LMH cells produce considerably more spliced than full-length pgRNA. Especially, the Sp1 spliced species is strongly increased while others are hardly visible in LMH cells. Furthermore, the CMV promoter enhances splicing levels not only in human HepG2 but also in chicken LMH cells. Mutational suppression of splicing, on the other hand, results in a significant increase of full-length pgRNA in the avian cell line. Comparing viral RNA from cytoplasm with encapsidated RNA from immunoprecipitated capsids indicated that relative packaging of spliced molecules is similar in HepG2 and LMH cells (see Figure S1). Therefore, high levels of spliced DNA in HBV capsids from LMH cells are primarily due to the enhanced splicing of viral RNA between genome position 547 and 1771.

Next, we prepared viral DNA from transfected HepG2 and LMH cells, and we performed PCR analysis using primers spanning the intron sequences. In HepG2 cells, splice-specific amplification products were detectable only upon transfection of splicing-competent HBV and CMV promoter plasmids but not in case of splice-deficient HBV promoter plasmid transfection (Figure 2B, left panel). In LMH cells, very strong signals were obtained for splice-derived products upon transfection of splicing competent plasmids, and those signals were absent in case of splice-deficient plasmid transfection (Figure 2B, right panel). Hence, the single A1769C mutation efficiently suppressed pgRNA splicing and subsequent synthesis of viral DNA from spliced transcripts in avian and human cells.

Furthermore, we performed Southern blot analysis to visualize directly viral DNA made in the respective capsids. In HepG2 cells, no major difference in quantity and pattern of replicative DNA was seen upon transfection with splice-deficient versus splicing-competent HBV promoter plasmid (Figure 2C, left panel). Use of the CMV promoter plasmid enhanced replication levels in HepG2 cells, but the pattern of viral DNA remained largely unchanged. In LMH cells, splice-derived DNA was clearly detectable upon transfection of splicing-competent HBV and CMV promoter plasmids. The corresponding signal was absent from cells that were transfected with the splice-deficient construct (Figure 2C, right panel). Surprisingly, much of the viral DNA from cytoplasm LMH cells, that were transfected with splice-deficient HBV promoter plasmid, migrated between the 3.2 kb and 2.0 kb marker fragments (see arrow in Figure 2C, right panel). Such DNA is also detectable in case of splicing-competent plasmid transfection, but becomes evident more clearly in the absence of splicing.

To facilitate quantitative assessment of the various DNA species, we denatured aliquots of each sample prior to electrophoresis. Thus, double strand mature genomes and single stranded precursor molecules will migrate at the same position in the gel. Subsequent Southern blot hybridisation was done with forward oligonucleotides binding close to the 3′-end of minus strand DNA in order to visualize spliced and full-length genomes with equal efficiency. As shown in the left panel of Figure 2D, HBV promoter plasmid transfected HepG2 cells predominantly produce full-length genomes and the spliced genomes are scarcely visible because of their low abundance. The spliced genomes, however, are clearly detectable when instead the CMV promoter construct was used. In LMH cells, by comparison, very strong signals are seen for the spliced genomes, especially in case of CMV promoter plasmid transfection (Figure 2D, right panel).

Quantitation of the Southern blot signals by phosphor imager revealed that the CMV promoter enhances synthesis of full-length DNA by a factor of three in human HepG2 cells. The full-length genomes are about three times more abundant than the spliced genomes in CMV promoter plasmid transfected HepG2 cells. In LMH cells, this ratio is reversed, with about two-times more spliced than full-length genomes for the HBV promoter construct, and about five-times more spliced than full-length genomes for the CMV promoter construct. Mutation of the splice-acceptor site, on the other hand, increases the amount of full-length genomes by a factor of five in the avian cell line. Altogether, these results demonstrate that enhanced splicing of pgRNA in LMH cells gives rise to increased amounts of spliced DNA. High level of splicing in chicken LMH cells, on the other hand, reduces the amount of full-length pgRNA and thereby diminishes the number of viral particles replicating the full-length HBV genome.

### HBV capsids from cytoplasm of LMH cells contain shortened viral genomes

**Figure 3:**
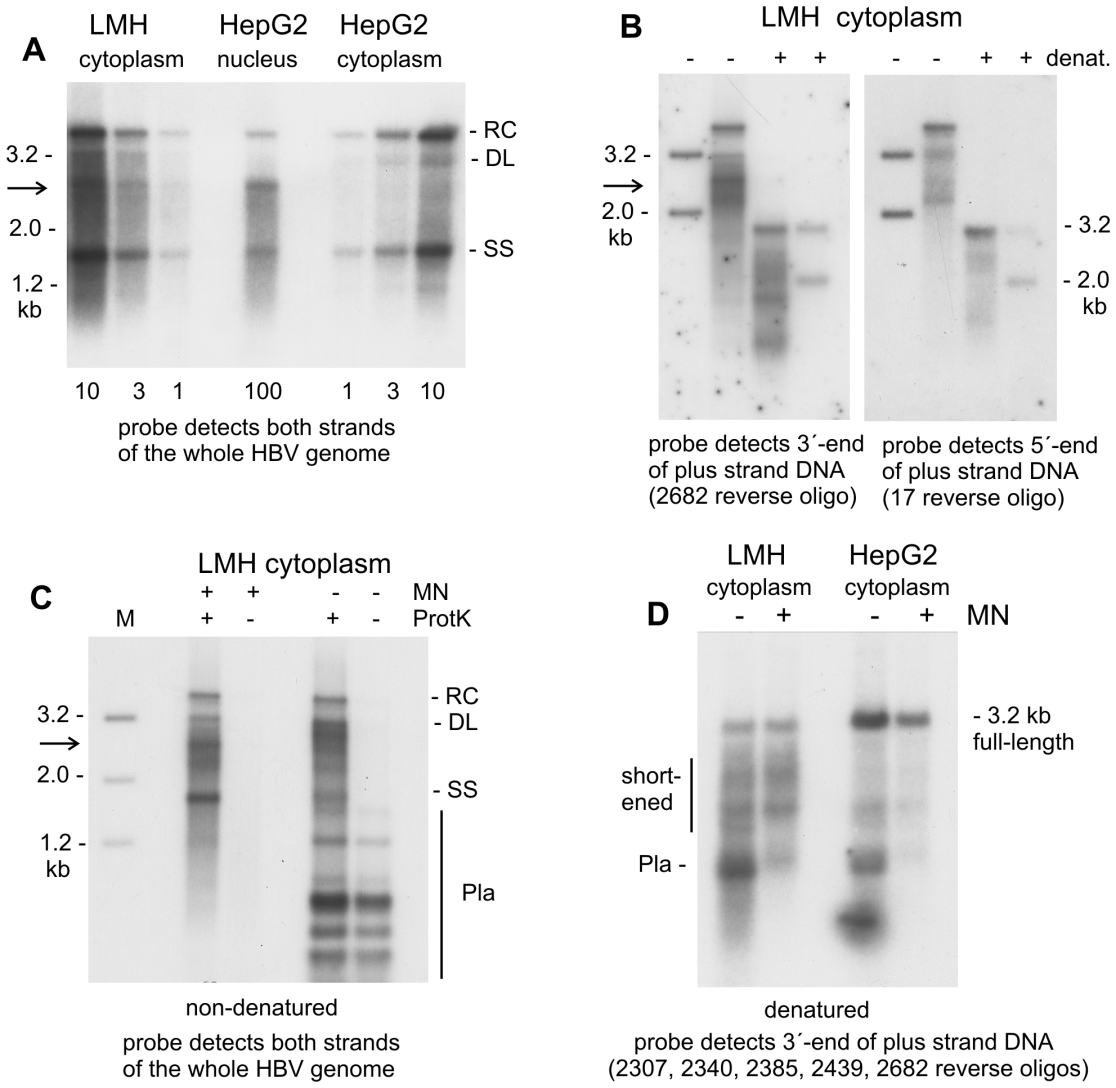
Detection of shortened HBV genomes in the cytoplasm of LMH cells. Transfection was done with a plasmid coding for splice-deficient virus with the natural HBV core promoter driving transcription of pgRNA and the surface protein coding sequence mutated. (**A**) Viral DNA from cytoplasm of LMH cells was compared with viral DNA from HepG2 cell nuclei and HepG2 cytoplasm. Preparation of viral DNA involved nuclease treatment of cytoplasmic lysates and purified cell nuclei. Numbers below indicate relative amounts of DNA loaded in the respective lanes. Hybridisation was done with a probe encompassing both strands of the whole HBV genome. The arrow points to a prominent DNA species obtained both in cytoplasm of LMH cells and nucleus of HepG2 cells. (**B**) Viral DNA from transfected LMH cells was denatured (+/− denat) by heating before gel loading, as indicated. Hybridisation was done with ^32^P-labelled oligonucleotides detecting either the 3′-end or the 5′-end of plus-strand DNA at genome positions 2682 and 17 (see map of the HBV genome in Figure 1D). Positions of the 3.2 kb and 2.0 kb marker fragments are indicated. The arrow points to viral genomes migrating between these marker fragments. (**C**) Viral DNA was prepared from cytoplasm of transfected LMH cells with or without micrococcal nuclease (+/− MN) treatment and with or without protease (+/− ProtK) digestion done before phenol extraction. The purified DNA was digested with restriction enzyme *Dpn*I, which selectively cuts transfected plasmid (Pla) into multiple short fragments. Southern blot hybridisation was done with a probe covering both strands of the whole HBV genome. This probe also detects several *Dpn*I fragments of transfected plasmid DNA. (**D**) Viral DNA was prepared from cytoplasm of HepG2 and LMH cells with or without micrococcal nuclease (+/− MN) treatment. Purified DNA was subsequently digested with restriction enzyme *Dpn*I and denatured by heating before gel electrophoresis. Southern blot hybridisation was done with a mixture of ^32^P-labeled oligonucleotide complementary to the 3′-end of plus-strand DNA at genome position 2307, 2340, 2385, 2439 and 2682. These five oligonucleotides were combined to enhance sensitivity. The vertical lane on the right side indicates shortened plus-strand molecules. The probe detects a 0.8 kb *Dpn*I fragment of the transfected plasmid (Pla) if micrococcal nuclease treatment is not done.

As mentioned above, much of the DNA present in viral particles from LMH cells migrates between the 3.2 kb and 2.0 kb marker fragments (see arrow in the right panel of Figure 2C). Such migration pattern might be due to incomplete elongation of plus-strand DNA molecules, as commonly seen with HBV particles from human blood. Alternatively, the characteristic profile may relate to the absence of specific DNA sequences as recently observed with HBV capsids from the nucleus of HepG2 cells [17]. As shown in Figure 3A, the most prominent DNA species in HBV capsids from HepG2 cell nucleus display the same electrophoretic mobility as those DNA molecules from cytoplasm of LMH cells that migrate between the 3.2 kb and 2.0 kb marker fragments. We have recently reported that HBV genomes from nucleus of HepG2 cells lack sequences in the core protein coding region, and these sequences may also be absent in viral DNA from cytoplasm of LMH cells.

To test for this hypothesis, we visualized viral DNA from splice-deficient HBV plasmid transfected LMH cells by Southern blot hybridisation with oligonucleotides binding near the 3′-end and near the 5′-end of plus-strand DNA, respectively. As expected, both probes detect rcDNA which co-migrates, after heating, with the 3.2 kb denatured marker fragment (Figure 3B). This rcDNA is derived from capsids containing completely mature, full-length HBV genomes. The 3′-end specific probe additionally detects DNA migrating between the 3.2 kb and 2.0 kb marker fragments (see arrow in left panel of Figure 3B). Yet, such DNA is scarcely visible with the 5′-end specific probe, which instead visualizes a faint smear of signals in the corresponding area (Figure 3B, right panel). Denaturation of the DNA samples before gel electrophoresis revealed that most of the plus-strand molecules that are detected with the 3′-end specific probe, are heterogeneous in size with some of them being shorter than 2.0 kb (Figure 3B, left panel). As the probe binds to the 3′-end, the respective plus-strand molecules must lack sequences at the 5′-end, which corresponds to the core protein coding region.

We speculated that the lack of sequence in the core coding region might be due to selective degradation of the respective region in the HBV genome during DNA preparation, which involves nuclease treatment of viral capsids. To test for this possibility, we prepared viral DNA with and without micrococcal nuclease treatment of cytoplasmic lysates. In addition, we asked whether the polymerase protein is linked with the viral genome. For this reason, protease treatment was omitted with an aliquot of each sample, which results in partitioning of protein-linked nucleic acid into the organic phase during phenol extraction and therefore loss of the respective genomes during DNA preparation.

Preparation of viral DNA from splice-deficient HBV plasmid transfected LMH cells by the standard procedure, which includes nuclease treatment of cytoplasmic lysate and subsequent protease digestion, resulted in the typical pattern of DNA with a prominent signal migrating between the 3.2 kb band 2.0 kb marker fragments (see arrow in Figure 3C). These signals are much weaker when protease treatment was omitted before phenol extraction, indicating that viral genomes that are protected inside the capsid are covalently linked with polymerase protein. Omitting micrococcal nuclease treatment before protease digest and phenol extraction resulted in a slight upward shift of the major signal close to the position of the 3.2 kb marker fragment. Therefore, the respective HBV genomes were not properly protected in viral capsids and were at least partially accessible to nuclease digestion. Omitting additionally the protease digest strongly diminished the signals close to the 3.2 kb marker fragment. Thus, those genomes that are not properly protected in viral capsids, are nevertheless linked with polymerase protein. HBV genomes migrating close to the 3.2 kb marker fragment may have been converted into shortened genomes during nuclease treatment because of partial degradation. Alternatively, the nuclease enzyme may have selectively removed those species migrating close to the 3.2 kb linear marker fragment, rendering the shortened genomes more clearly visible.

To distinguish between these two alternatives, we prepared viral DNA from cytoplasm of LMH cells with or without nuclease treatment. The purified DNA was denatured before gel electrophoresis and subsequently visualized by hybridisation with oligonucleotides binding to the 3′-prime end of plus-strand DNA. In this experiment, we also included viral DNA from cytoplasm of HepG2 cells, for reference. As seen in Figure 3D, there are various shortened plus-strand molecules visible that migrate clearly faster than 3.2 kb full-length DNA. These plus-strand molecules are present both in the micrococcal nuclease treated and in the non-treated sample. Thus, the shortened genomes are most likely not generated during nuclease treatment. Instead, they are a priori present in HBV capsids from cytoplasm of LMH cells. Remarkably, shortened genomes are also detectable in denatured viral DNA from cytoplasm of HepG2 cells. However, the corresponding signals are very weak compared to the signal of 3.2 kb full-length genomes and compared to the respective signals in LMH cells.

## Discussion

Splicing occurs in most and potentially all natural HBV variants. Yet, the importance of splicing during HBV infection remains elusive [24]. Usually, less than 10% of the viral DNA genomes in human serum derived HBV particles are modified by splicing [25], [26]. HBV particles containing spliced genomes probably do not contribute to virus amplification, because essential protein coding sequences are lost during splicing. Therefore, splicing is of potential disadvantage for virus reproduction and needs to be kept at tolerable levels in the infected liver cells.

Spliced RNA is also made in human hepatoma cells that are transfected with HBV coding plasmids [22], [27]. With the natural HBV core promoter, the amount of spliced RNA is significantly less than that of full-length pgRNA. Transfection of CMV promoter plasmids, however, increases the splicing levels and spliced transcripts are present at slightly higher amount than full-length pgRNA. Nevertheless, there are clearly less spliced than full-length DNA genomes present in viral capsids from CMV promoter plasmid transfected HepG2 cells. The apparent discrepancy in the relative levels of spliced RNA and DNA can be explained by the fact that HBV polymerase preferentially binds the full-length pgRNA from which the protein was translated [28]. Thus, the polymerase favours encapsidation of full-length pgRNA, provided that both transcripts arise from the same template. However, spliced and full-length pgRNA have equal chances to enter viral capsids if the polymerase coding RNA and the pgRNA to be encapsidated are transcribed from two different plasmids. Therefore, up to 50% of viral DNA is derived from spliced pgRNA if human hepatoma cells are co-transfected with CMV promoter plasmids, as previously reported by Abraham *et al.*, 2008.

Most studies on HBV replication in human hepatoma cells do not pay specific attention to the spliced DNA molecules because they are usually not visible in the background of signals from nascent full-length genomes. Detection of spliced genomes, however, needs to be taken into account if *trans*-complementation experiments are done and if CMV promoter plasmids are applied. In addition, the spliced DNA becomes especially evident with specific mutations in the carboxyterminal domain of the HBV core protein. The phenotype of these mutants is due to destabilisation of full-length pgRNA containing capsids and selective synthesis of short genomes derived from spliced transcripts [29], [30]. This phenomenon was initially discovered with CMV promoter plasmids and was not noticed in other studies, presumably because HBV promoter plasmids were applied in the respective investigations [31], [32], [33].

Notably, complete absence of viral genomes derived from full-length pgRNA has only been observed so far, if nearly half of the carboxyterminal domain of the HBV core protein was deleted or if three serine phosphorylation sites in the carboxyterminal domain were collectively mutated [29], [30]. Other mutants displayed an intermediary phenotype with some tendency to get less full-length genomes than with wild type. This phenomenon is generally thought to reflect a role of serine phosphorylation and dephosphorylation during elongation of viral DNA [34]. However, preferential synthesis of shortened genomes, like those abundantly present in LMH cells, may also explain the phenotype. Thus, it might be revealing to compare the replication of HBV core mutants in HepG2 and LMH cells to understand more clearly the effects of mutations in the carboxyterminal domain on synthesis of viral DNA. In addition, it might be worthwhile to examine more extensively the core protein made in LMH cells for the presence of post-translational modifications, which probably requires mass spectrometry, as protein gel electrophoresis does not disclose any migration differences in comparison with HBV core protein from HepG2 cells (see Figure S2).

High abundance of spliced genomes in HBV capsids from transfected LMH cells is primarily due to excessive splicing of pgRNA. Especially, the Sp1 spliced species is strongly enhanced in the avian cell line. Other spliced transcripts, like the Sp3 and Sp14 species, are also made but they are not profoundly increased. HBV replication in LMH cells clearly illustrates that the synthesis of spliced genomes occurs at the expense of full-length genomes. In human HepG2 cells, such competition is less evident and the splicing process remains at low enough levels to permit sufficient virus production. Nonetheless, it is puzzling why splicing of HBV transcripts happens at all. Our investigation shows that splicing can be abolished with a single nucleotide exchange. Notably, the A1769C mutation, examined in this study, has no effect on viral protein sequences. Therefore, one would expect that an equivalent mutation occurs as a common variant in HBV infected patients. Most HBV sequences deposited to GenBank, however, possess the splice acceptor site with an adenine at position 1769. The presence of splice sites in natural virus variants strongly argues for an important role of splicing during HBV infection, and exploring the relevance of this process in the HBV life cycle will be a major challenge of future research.

Splicing also occurs with DHBV transcripts. However, the splice sites in the DHBV genome localize to positions different from those in the HBV genome [35]. Nevertheless, splicing of DHBV transcripts also disrupts the polymerase coding sequence and reduces the amount of full-length pgRNA available for synthesis of infectious viruses. Interestingly, a secondary structure, caused by complementary sequences near the splice donor and acceptor sites, protects DHBV pregenomic RNA from excessive splicing [36]. Such complementary sequences are not evident in the HBV genome and it is not yet known whether other control mechanisms exist that potentially down-regulate splicing of HBV transcripts in human cells.

Increased splicing of HBV transcripts in LMH cells may simply be due to better conformance of the avian spliceosome with the respective sequences in the HBV genome, in particular regarding the Sp1 splice donor site. However, the 5′-end sequences of U1 small nuclear RNA, which recognize splice donor sites, are identical in human and chicken [37]. Alternatively, a specific control mechanism that down-regulates splicing of HBV transcripts in human cells might be absent in avian cells. Future studies, comparing RNA synthesis in HepG2 and LMH cells, may help to reveal a control mechanism that down-regulates splicing of HBV transcripts in the natural host. Of note, the prevention of excessive splicing may represent a hitherto little-thought-of factor restricting the species tropism of HBV.

We have recently shown that DHBV replicates in human HepG2 cells with the same pattern and efficiency as in chicken LMH cells. Human HBV, on the other hand, is able to replicate in chicken LMH cells, but here the viral DNA looks different from viral DNA obtained in HepG2 cells. The distinct pattern of viral DNA is primarily due to enhanced splicing of pgRNA in the avian cell line. Furthermore, many of the HBV capsids from cytoplasm of transfected LMH cells contain shortened genomes that lack sequences in the core protein coding region. So far, it is not clear how the shortened genomes arise and why they are so abundant in chicken LMH cells. One possible explanation for the observed absence of DNA sequences in the core protein coding region is premature stop of minus-strand elongation and subsequent start of plus-strand synthesis from RNA fragments that were not fully digested by the RNase H activity of HBV polymerase. Remarkably, shortened genomes can also be found in cytoplasm of HepG2 cells. However, they are present there at very low abundance, and for this reason probably remained undetected until now.

Furthermore, there are capsids in the cytoplasm of LMH cells that do not protect the viral genome. The corresponding molecules migrate slightly beneath 3.2 kb double strand linear indicating that they represent genomes in which plus-strand synthesis has initiated upstream of the core protein reading frame or at least the complementary minus-strand is fully elongated. These genomes are lost if DNA preparation includes nuclease treatment of cytoplasmic lysate. So far, it is not clear whether non-protected genomes also exist in cytoplasm of HepG2 cells. At least, omission of nuclease treatment does not make an obvious difference in the pattern of viral DNA obtained from cytoplasm of human HepG2 cells (see Figure 1B in Köck *et al.*, 2010).

Nuclease treatment, however, makes a clear-cut difference regarding the viral DNA obtained from the nucleus of HepG2 cells: HepG2 cell nuclei, first, contain rcDNA that is released from the capsid and detached from the polymerase protein. Such rcDNA molecules are digested if HepG2 cell nuclei are treated with micrococcal nuclease enzyme. Second, there are viral genomes that migrate close to 3.2 kb double strand linear DNA. These genomes are likewise not protected from nuclease digestion but they are still connected with polymerase protein (see Figure 7A, right panel in Köck *et al.*, 2010). Third, there are genomes that migrate clearly faster than 3.2 kb double strand linear, are protected in viral capsids and are connected with polymerase protein (see Figure 7B, right panel in Köck *et al.*, 2010). We previously speculated that the faster migrating species arise from full-length genomes that were partially digested in the core protein coding region during micrococcal nuclease treatment of HepG2 cell nuclei. The here presented data, however, indicate that such genomes also exist in cytoplasm of HepG2 cells, and that they are present even if nuclease treatment is not done. Thus, they are most likely not generated during nuclease treatment of HepG2 cell nuclei. Instead, they become clearly visible because other HBV genomes that are not protected in viral capsids are removed by the nuclease enzyme.

Interestingly, incomplete protection of viral genomes in HepG2 cell nuclei is found only if HBV surface protein is absent (see Figure S3). Chicken LMH cells do not synthesize HBV surface RNA and thus do not produce surface proteins. So far, we have not yet tested whether the presence of surface protein potentially makes a difference regarding nuclease sensitivity of viral genomes in HBV capsids from cytoplasm of LMH cells. This question will be addressed in future studies by co-transfection of appropriate expression plasmids coding for HBV surface proteins.

The cell nucleus is the place where the HBV capsid needs to open for release of rcDNA and subsequent cccDNA formation. Until now, almost nothing is known about the biochemical events that trigger HBV capsid disassembly in the cell nucleus. The here presented data indicate, that a process potentially reflecting capsid disassembly in the nucleus of human HepG2 cells takes place in cytoplasm of chicken LMH cells. In particular, the incomplete protection of viral genomes migrating close to 3.2 kb double strand linear may mimic the opening of HBV capsids in the cell nucleus. Studies of HBV capsid disassembly in HepG2 cell nuclei are limited by the low number of viral particles present there. Cytoplasm of LMH cells, on the other hand, abundantly contains apparently opened capsids facilitating large-scale preparation for more extensive biochemical and structural analysis. Thus, transfection of LMH cells with HBV coding plasmids may pave the way for follow-up investigations on the mechanism of capsid disintegration which is an essential prerequisite for cccDNA formation during infection and intracellular recycling of viral genomes.

## Supporting Information

Figure S1 **HepG2 and LMH cells were transfected with HBV coding plasmid having the polymerase sequence mutated (YMHD instead of YMDD which results in deficiency for reverse transcriptase activity).** The RNA was prepared from total cytoplasm and from HBV capsids that were obtained by immunoprecipitation. Immunoprecipitation was done from 300 µl cytoplasmic lysate, whereas total RNA isolation was done from 100 µl cytoplasmic lysate. The probe detects the 5′-end of spliced and full-length pgRNA. The 18S and the 28S ribosomal RNAs served as size markers. Note that the relative efficiencies of spliced and full-length pgRNA packaging are similar in HepG2 and LMH cells.
(TIF)

Figure S2 **HepG2 and LMH cells were transfected with wild type HBV and with GFP coding plasmids.** The GFP protein and the HBV core protein were visualized by Western blot analysis. Numbers below indicate relative amounts of cell lysate loaded in the respective lane. n.t. is a non-transfected control. Positions of size marker proteins (17 and 25 kDa) are shown on the left side. Note that HBV core proteins from HepG2 and LMH cells migrate at the same position in the gel. Thus, there is no evidence of differing post-translational modification in both cell lines, yet. The HBV core protein is translated from full-length pgRNA and from spliced RNA causing high abundance of core protein in LMH cells.
(TIF)

Figure S3 **HepG2 and LMH cells were transfected with splice-deficient HBV coding plasmid, in which the surface-coding sequence was either mutated (surface-negative) or wild type (surface-positive).** Viral DNA was prepared from micrococcal nuclease treated cytoplasm and from micrococcal nuclease treated cell nuclei. Numbers below indicate relative amounts of DNA loaded. The probe applied detects both strands of the whole HBV genome. Note that cytoplasm of surface-positive HBV producing cells harbours less rcDNA containing capsids than cytoplasm of surface-negative HBV producing cells; probably because rcDNA containing capsids are secreted into culture supernatant if surface protein is present. Most interesting, viral capids in the nucleus of surface-positive HBV producing cells protect all genome species, whereas capsids in the nucleus of surface-negative HBV producing cells fail to protect mainly the full-length genomes from micrococcal nuclease digestion.
(TIF)
