# Supplementary figures and images for: Human Hepatitis B Virus Production in Avian Cells Is Characterized by Enhanced RNA Splicing and the Presence of Capsids Containing Shortened Genomes
Source: PLoS One. 2012 May 18;7(5):e37248. doi: 10.1371/journal.pone.0037248 (PMC3356268; doi:10.1371/journal.pone.0037248)

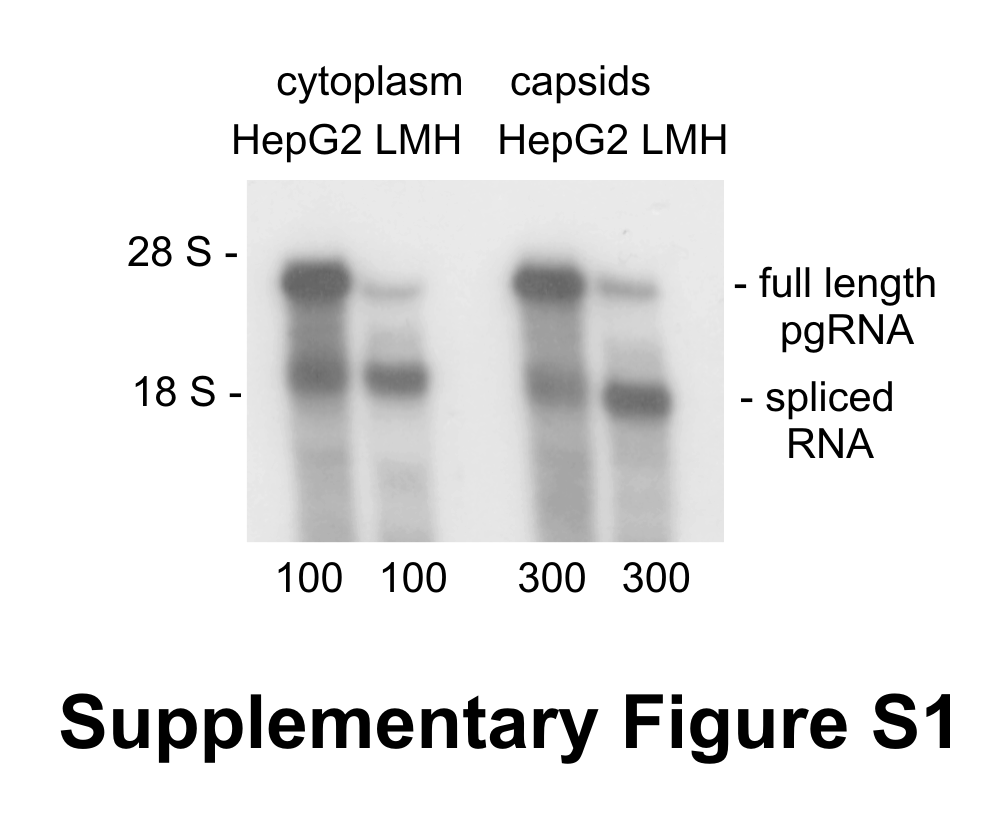

Supplement: Figure S1 — HepG2 and LMH cells were transfected with HBV coding plasmid having the polymerase sequence mutated (YMHD instead of YMDD which results in deficiency for reverse transcriptase activity). The RNA was prepared from total cytoplasm and from HBV capsids that were obtained by immunoprecipitation. Immunoprecipitation was done from 300 µl cytoplasmic lysate, whereas total RNA isolation was done from 100 µl cytoplasmic lysate. The probe detects the 5′-end of spliced and full-length pgRNA. The 18S and the 28S ribosomal RNAs served as size markers. Note that the relative efficiencies of spliced and full-length pgRNA packaging are similar in HepG2 and LMH cells. (TIF) [file pone.0037248.s001.tif]

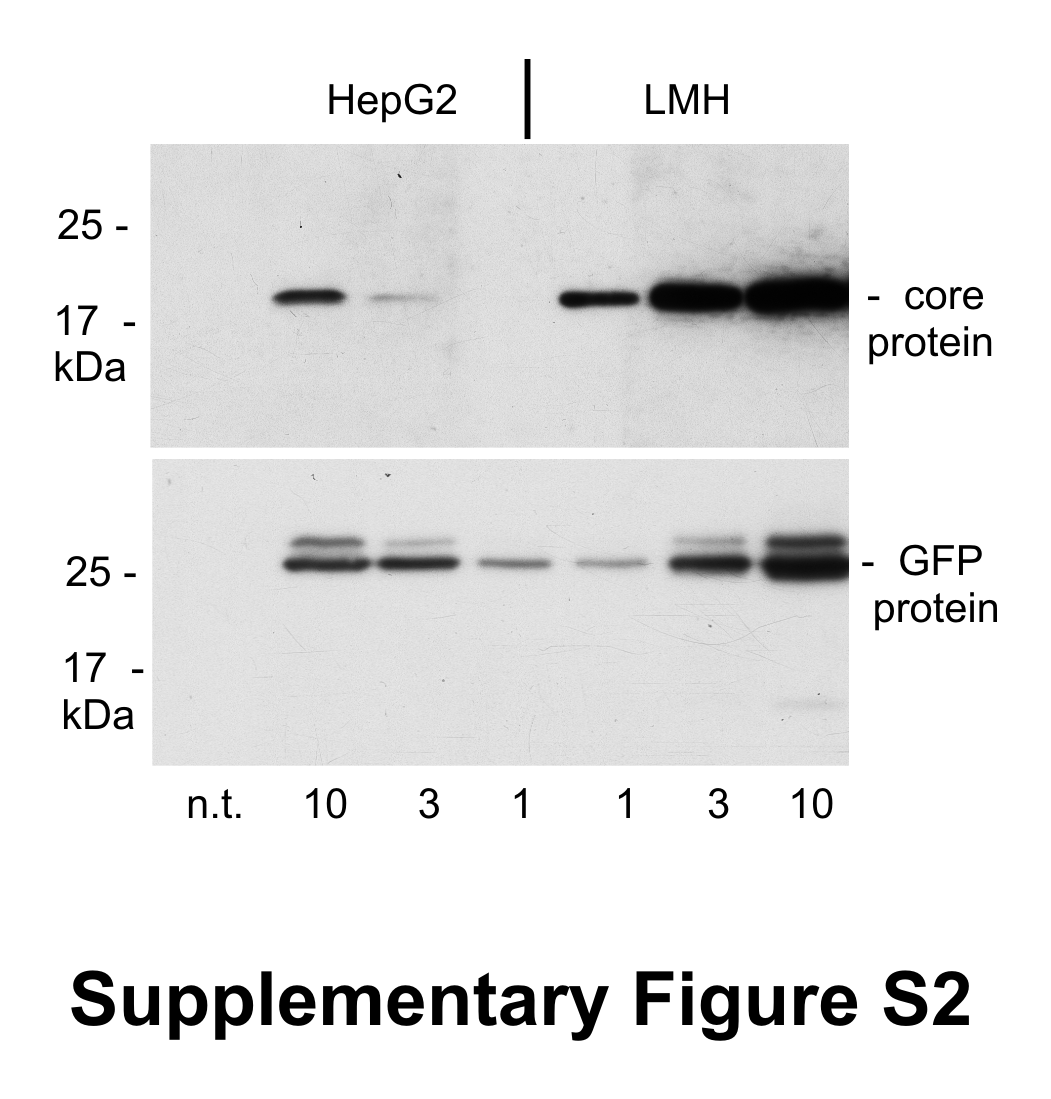

Supplement: Figure S2 — HepG2 and LMH cells were transfected with wild type HBV and with GFP coding plasmids. The GFP protein and the HBV core protein were visualized by Western blot analysis. Numbers below indicate relative amounts of cell lysate loaded in the respective lane. n.t. is a non-transfected control. Positions of size marker proteins (17 and 25 kDa) are shown on the left side. Note that HBV core proteins from HepG2 and LMH cells migrate at the same position in the gel. Thus, there is no evidence of differing post-translational modification in both cell lines, yet. The HBV core protein is translated from full-length pgRNA and from spliced RNA causing high abundance of core protein in LMH cells. (TIF) [file pone.0037248.s002.tif]

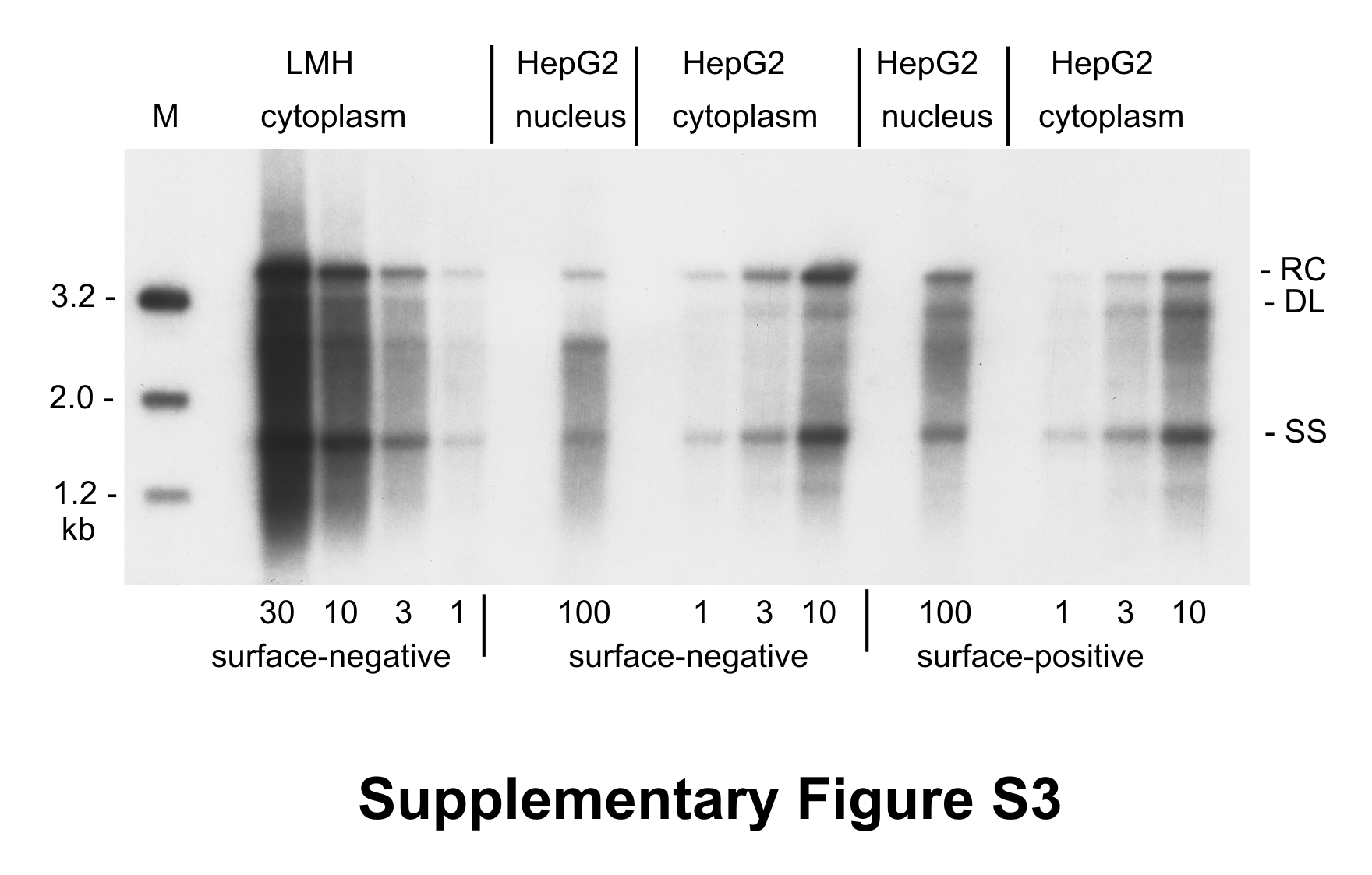

Supplement: Figure S3 — HepG2 and LMH cells were transfected with splice-deficient HBV coding plasmid, in which the surface-coding sequence was either mutated (surface-negative) or wild type (surface-positive). Viral DNA was prepared from micrococcal nuclease treated cytoplasm and from micrococcal nuclease treated cell nuclei. Numbers below indicate relative amounts of DNA loaded. The probe applied detects both strands of the whole HBV genome. Note that cytoplasm of surface-positive HBV producing cells harbours less rcDNA containing capsids than cytoplasm of surface-negative HBV producing cells; probably because rcDNA containing capsids are secreted into culture supernatant if surface protein is present. Most interesting, viral capids in the nucleus of surface-positive HBV producing cells protect all genome species, whereas capsids in the nucleus of surface-negative HBV producing cells fail to protect mainly the full-length genomes from micrococcal nuclease digestion. (TIF) [file pone.0037248.s003.tif]
